# Supplementary material for: Allogenic faecal microbiota transplantation for antibiotic-associated diarrhoea in critically ill patients (FEBATRICE)–Study protocol for a multi-centre randomised controlled trial (phase II)
Source: PLoS One. 2024 Dec 27;19(12):e0310180. doi: 10.1371/journal.pone.0310180 (PMC11676529; doi:10.1371/journal.pone.0310180)
Supplement: S1 Appendix — (DOCX) [file pone.0310180.s001.docx]

**Donor Management and Faecal Microbiota Tranplantate preparation and storage**

**Donor selection**

The donor should generally be a healthy person aged 18-60 years, selected on voluntary participation in the study, fulfillment of all necessary conditions as inclusion and exclusion criteria (see below), exclusion of risk factors (see questionnaire), health status determined by physical examination and laboratory tests (see below), negative results of screening for intestinal pathogens in stool and swabs. The aim of the basic blood test is to confirm the patient's good clinical condition. The aim of other tests is to prevent the transmission of latent infection from the donor to the recipient. This complete examination of the donor is repeated after 2 months. If the following examination is also found to be physiological (i.e. without signs of the donor's infectious disease), the stool donated by the donor during these 2 quarantine months (frozen in the stool bank) will be considered non-infectious and can be used for the FBT method. Despite this quarantine measure, the donor is obliged to fill in a questionnaire focusing on the risks of infectious diseases during each delivery of stool. In case of a new occurrence of infectious manifestations (especially diarrhea) travelled abroad or taken antibiotics the stool donor is obliged to inform about this attending physician.

**The questionnaire** contains a list of questions aimed at detecting risk behavior, risk of infectious diseases or exclusion criteria related to the medical history of potential donors (see Appendix 2- questionnaire). **Physical examination:** all donors will have their blood pressure and pulse taken and will have a complete physical examination (general condition, breathing, heart sounds, abdominal examination, etc.). If a donor is found suitable according to the questionnaire and physical examination, he/she is subjected to a comprehensive examination. ***Laboratory tests include***: **1. basic blood tests** (blood count + differential, CRP, ALT, AST, ALP, GMT, bilirubin, creatinine, glycaemia. **2. stool examination** for Clostridium difficile PCR, culture for common intestinal pathogens (Salmonella, Campylobacter, Shigella, Yersinia), enteropathogenic E. coli (EPEC,EAEC,ETEC,EIEC,STEC), Vibrio cholerae (if the donor has been in tropical areas in the last 6 months), Listeria, CMV, Norovirus, Rotavirus, ATB-resistant microorganisms (MDRO): VRE, ESBL, CRE. Antigen H. pylori from faeces, occult bleeding test. **3. stool parasitological examination** for standard pathogens and Giardinia lamblia, Cryptosporidium parvum, Isospora, Microsporidia, Entamoeba histolytica, Dientamoeba fragilis, Strongyloides stercoralis (PCR), Cyclospora and Isospora, Blastocystis hominis. **4. serological tests** - HIV 1,2 (antibodies to HIV 1,2, antigen test), syphilis (RRR or TPPA), screening for viral hepatitis A,B,C,E (anti-HAV IgM, HBsAg, anti-HCV, anti-HEV IgM). **5. nasopharyngeal swab** PCR SARS-CoV-2, MRSA.

Note: for rapid stool analysis, a Gastrointestinal (GI) panel can be used to detect common gastrointestinal pathogens including viruses, bacteria and parasites. The system integrates sample preparation, amplification, detection and analysis. Total testing time is 1h. In our hospital we use BIOFIRE® FILMARRAY® instruments (FDA, CE-IVD, TGA certified). Pre-prepared BIOFIRE® FILMARRAY®GI Panels are used for analysis. List of pathogens, BIOFIRE® FILMARRAY®GI Panel: Campylobacter (jejuni, coli & upsaliensis), Clostridium difficile (Toxin A/B), Plesiomonas shigeloides, Salmonella, Yersenia enterocolica, Vibrio (cholerea parahaemolyticus, vulnificus), E. coli (EAEC,EPEC,ETEC,STEC, EIEC), Adenovirus, Astrovirus, Norovirus, Rotavirus A, Sapovirus (I,II,IV,V), Cryptosporidium, Cyclospora cayetanensis, Entamoeba histolytica, Giardia lamblia

***Stool collection from the donor:***

For better timing of defecation, the donor will use glycerine suppository (Suppositoria Glycerini 2,06g). The stool will be captured via Fe-Col Fecal Collection paper (Eastoport) and transported in a sterile container (container 2000ml, Sarsted) to the place of processing as quickly as possible, but no later than 2 hours from defecation. To maintain optimal stool temperature during transport, the container can be transported in a thermal bag, especially during summer days when the outside temperature is above 30°C.

**Preparation of stool filtrate for FMT**. As the vast majority of bacteria in stool are anaerobic, a fresh stool sample from the donor must be processed as soon as possible and mixed with glycerol and frozen at -80stC for later use. Stool should be processed no later than 6 hours after defecation to avoid a decrease in viable bacteria, which are essential for the effect of FMT. In studies where this interval has been extended, the efficacy of FMT has subsequently been reduced[1,2]. After the donor has brought the stool, early processing of the stool must take place in a designated area (in consultation with the hospital hygienist) where sanitation of the premises and accessories can be carried out as recommended. Visual inspection of the stool is important before the actual processing[1]. Fresh stool from the donor should be free of diarrhea (Bristol Stool Score) and should not contain mucus or blood. If the specimen is judged suitable, homogenization of the fresh stool should be performed. Sterile saline is used for homogenization and the stool is diluted approximately 1:3-1:5, depending on the consistency. The density is important for the resulting transplant, and the mixing time, approximately 1-2 min, is also important. Homogenization is carried out using a mixer dedicated for this purpose only, with the possibility of optimal sanitation and sterilized after each use by a two-stage disinfection. The homogenized product is then filtered through gauze to remove undigested food residues and other undesirable impurities. The use of 2 layers of gauze seems optimal. If a smaller layer of gauze is used, there is an undesirable admixture of large indigestible residues; if, on the other hand, a higher layer is used, there is insufficient filtration and a large amount of unfiltered material is lost. To freeze the filtrate for later use, the resulting extract must be treated with a cryoprotective substance. For this purpose, according to recommendations, glycerol is used so that its concentration in the final product is about 10-15 %[1]. Glycerol prevents the formation of large ice crystals and thus protects bacteria from the effect of freezing.

**Quarantine and safety measures for frozen transplants**

The frozen stool sample must not be thawed and used for FBT for 2 months from the date of collection due to quarantine measures. The donor must inform the physician of any change in his/her health status (especially infectious disease, diarrhea, etc.) at any time since the donation of the stool. If the donor donates stool repeatedly, he/she should complete a current questionnaire each time he/she brings in stool and undergo a complete screening based on blood and stool analysis at least every 3 months[1]. At least 4 weeks after the last stool collection, the donor is again fully screened (physical examination, blood and stool tests identical to the initial screening) for possible false-negative serologic testing for infectious diseases. Pending evaluation of the results, the frozen stool is quarantined and cannot be used. Only when all samples collected are confirmed negative for infection can the donor be considered non-infectious and the frozen sample is considered safe and can be used for FBT purposes[1]. FBT stool suspension filtrate will be stored in specific −80°C freezer with connected alarm notification to guarantee continuous registration of the storage. It can be stored and subsequently used for a maximum of 12 months, as recommended according the stool bank guidelines, due to the decrease in live microbial counts with prolonged storage[1,3]. Information on the FBT suspension labels includes donor code, suspension number, production and expiration date, volume, and storage temperature instruction. From each donor one of the samples will be store in freezer for the duration of the ongoing study.

1. Keller JJ, Ooijevaar RE, Hvas CL, et al. (2021) A standardised model for stool banking for faecal microbiota transplantation: a consensus report from a multidisciplinary UEG working group. *United European Gastroenterol J* 9: 229–247.

2. Hota SS, Sales V, Tomlinson G, et al. (2017) Oral vancomycin followed by fecal transplantation versus tapering oral vancomycin treatment for recurrent clostridium difficile infection: An open-label, randomized controlled trial. *Clinical Infectious Diseases* 64: 265–271.

3. Cammarota G, Ianiro G, Kelly CR, et al. (2019) International consensus conference on stool banking for faecal microbiota transplantation in clinical practice. *Gut* 68: 2111–2121.
