# Supplementary material for: Allogenic faecal microbiota transplantation for antibiotic-associated diarrhoea in critically ill patients (FEBATRICE)–Study protocol for a multi-centre randomised controlled trial (phase II)
Source: PLoS One. 2024 Dec 27;19(12):e0310180. doi: 10.1371/journal.pone.0310180 (PMC11676529; doi:10.1371/journal.pone.0310180)
Supplement: S2 Appendix — (DOCX) [file pone.0310180.s002.docx]

QUESTIONNAIRE FOR DONORS

Faecal bacteriotherapy (faecal transplantation)

Patient (name, surname, date of birth, insurance company):


Department :

Doctor providing instruction: 


You have been selected as a suitable stool donor for a planned procedure called fecal bacteriotherapy (FBT). This is the transfer of stool from a healthy donor into the intestine of the recipient in order to restore the patient's (recipient's) disturbed intestinal flora. The information in the questionnaire is used to assess the health status and risk of possible infectious disease in the stool donor. Therefore, please fill in all data carefully and truthfully.

**Factors influencing the composition of gut bacteria**

- Weight............kg, Height...........cm, BMI (Body Mass Index)
- Do you personally or a family member work in healthcare? If so, do you live in the same household?

Yes No

- You have taken antibiotics or intestinal disinfectants (e.g. Endiaron) in the last 3 months. If yes, please specify which ones, the dose and the duration of use.

Yes No

…………………………………………………………………………….

- Are you taking medications that affect the immune system (corticosteroids, cyclosporine, immunosuppressants, biological therapy)? If yes, please specify which ones, the dose and the duration of use.

Yes No

……………………………………………………………………………..

- Are you allergic to anything? If yes, to what.

Yes No

- Do you take any medication regularly? If yes, what kind (name of medicine, dosage)

Yes No

……………………………………………….......................................

• Have you had chemotherapy or radiation?

Yes No

- Have you ever had a transplant? If yes, please indicate the date and what you had transplanted.

Yes No

……………………………………………………………………………….

• Are you being monitored for other diseases (diabetes mellitus, kardiac diseases, autoimmune diseases, etc.)? If yes, for which

Yes No

……………………………………………………………………………….

• Have you been vaccinated with a live vaccine (live virus, bacteria) in the last 3 months? If yes, which one and the exact date of administration?

Yes No

……………………………………………………………………………….

• Do you smoke? If yes, how many years and how many cigarettes a day?

Yes No

I have been smoking for ...........years, ......cigarettes/day

- Do you drink alcohol?
- I am abstinent
- I drink alcohol occasionally
- I consume alcohol regularly (min. 5 times a week)
- Approximately how many hours a day do you sleep?

................hrs/day

- Do you suffer from any of the following neurological diseases?
- Alzheimer's disease (AD) and other dementias
- Parkinson's disease (PD) and related disorders
- Prion diseases
- Motor neurone disease (MND)
- Huntington's disease (HD)
- Spinocerebellar ataxia (SCA)
- Spinal muscular atrophy (SMA)
- Other: ................................................

• Do you have medically confirmed depression? If so, what medication are you taking, the dose and time interval of the doses.

Yes No

……………………………………………….

**Diseases of the digestive tract**

- Are you or have you been treated by a gastroenterologist for ulcerative colitis, Crohn's disease, irritable bowel disease?

Yes No

- Have you had surgery on the digestive tract - surgery on the intestines, rectum, gallbladder, pancreas, liver, etc. If yes, how.

Yes No

……………………………………………………………………………..

- Are you being treated for another digestive tract disease? If yes, for which one.

Yes No

………………………………………………………………………………..

• Have you been treated for an gastrointestinal cancer?

Yes No

• Have you been treated for a parasitic infection or infection with Giardia lamblia and other microorganisms affecting the digestive system? If yes, please give the exact name of the infectious agent, date of onset, date of cessation, exact name of the medication you were taking and date of cessation of treatment.

Yes No

………………………………………………………………………………..

• Were you found to have polyps or diverticula (wall abnormalities) on your bowel during the colonoscopy?

Yes No

- Have you been examined in the past for gastrointestinal problems (constipation, diarrhea, blood in stool, etc.)? If yes, for what.

Yes No

**Infectious diseases**

- Have you had a febrile illness in the last 4 weeks or had symptoms such as abdominal cramps, diarrhea, enlarged lymph nodes?

Yes No

- Is anyone in your family experiencing an infectious disease at this time? (respiratory, diarrhea, other)? If yes, which disease.

Yes No

- Have you had a Covid-19 disease in the last 3 months?

Yes No

- Is anyone in your family currently on Covid-19?

Yes No

- In the past 4 weeks, have you met anyone who has been diagnosed with Covid-19 infection?

Yes No

- Have you been hospitalized recently?

Yes No

- In the past 6 months, have you been treated in conditions of poor hygiene? If yes, please indicate where and when (exact date of treatment)

Yes No

………………………………………………………………………………..

- Have you ever had an infection with multi-drug resistant organisms (MRSA, ESBL E.coli)?
  Yes No
- Have you ever been identified as a carrier of any infectious agent, including multidrug-resistant organisms?
  Yes No
- Have you received a transfusion, frozen plasma, blood or plasma derivatives in the last 6 months? 
  Yes No
- Are you being treated or monitored for hepatitis A, B, C, E or HIV/AIDS infection
  Yes No

• Have you been in contact with a patient with proven infectious hepatitis, HIV/AIDS?

Yes No

- Have you had close contact with a person at high risk of sexually transmitted infections? 
  (e.g. prostitute, homosexual, drug addict)
   Yes No
- Have you had a new sexual partner in the last 6 months?
  Yes No
- Are you a drug user?
   Yes No
- Have you had tattoos, piercings, earrings, acupuncture in the last 6 months?
  Yes No
- Have you been pricked with a potentially infectious needle in the last 6 months?

Yes No

- Have you travelled to an area with reduced hygiene standards in the last 6 months? (Mediterranean islands, Africa, Asia, Central America)

Yes No

I, the undersigned, declare that I have been clearly informed by my doctor about the method for which the biological material of which I am a donor will be used. The information and instructions have been communicated and explained to me by the doctor, I have understood them and have had the opportunity to ask additional questions, which have been answered by the doctor.
I also declare that I have given the doctor all the above information truthfully and that I have not concealed any reasons known to me that might have made the procedure difficult (in particular, medications taken, allergies, associated diseases, habits and contact with infectious disease).

In Prague, on: Signature of the donor:
