# Supplementary material for: Allogenic faecal microbiota transplantation for antibiotic-associated diarrhoea in critically ill patients (FEBATRICE)–Study protocol for a multi-centre randomised controlled trial (phase II)
Source: PLoS One. 2024 Dec 27;19(12):e0310180. doi: 10.1371/journal.pone.0310180 (PMC11676529; doi:10.1371/journal.pone.0310180)
Supplement: S3 Appendix — (DOCX) [file pone.0310180.s003.docx]

Biological Specimens (#33)

During the course of the study, plasma and stool samples will be collected from all participating patients. These samples will be subjected to analysis and then preserved at a temperature of -80°C for future exploratory studies as previously detailed. The faecal microbiome of both donors and patients will be identified using the quantitative sequence-based mapping technique. It's important to note that there are no intentions to conduct molecular analyses on human DNA. Once all required analyses are completed, the intention is to safely dispose of all samples.
