# Supplementary figures and images for: Allogenic faecal microbiota transplantation for antibiotic-associated diarrhoea in critically ill patients (FEBATRICE)–Study protocol for a multi-centre randomised controlled trial (phase II)
Source: PLoS One. 2024 Dec 27;19(12):e0310180. doi: 10.1371/journal.pone.0310180 (PMC11676529; doi:10.1371/journal.pone.0310180)

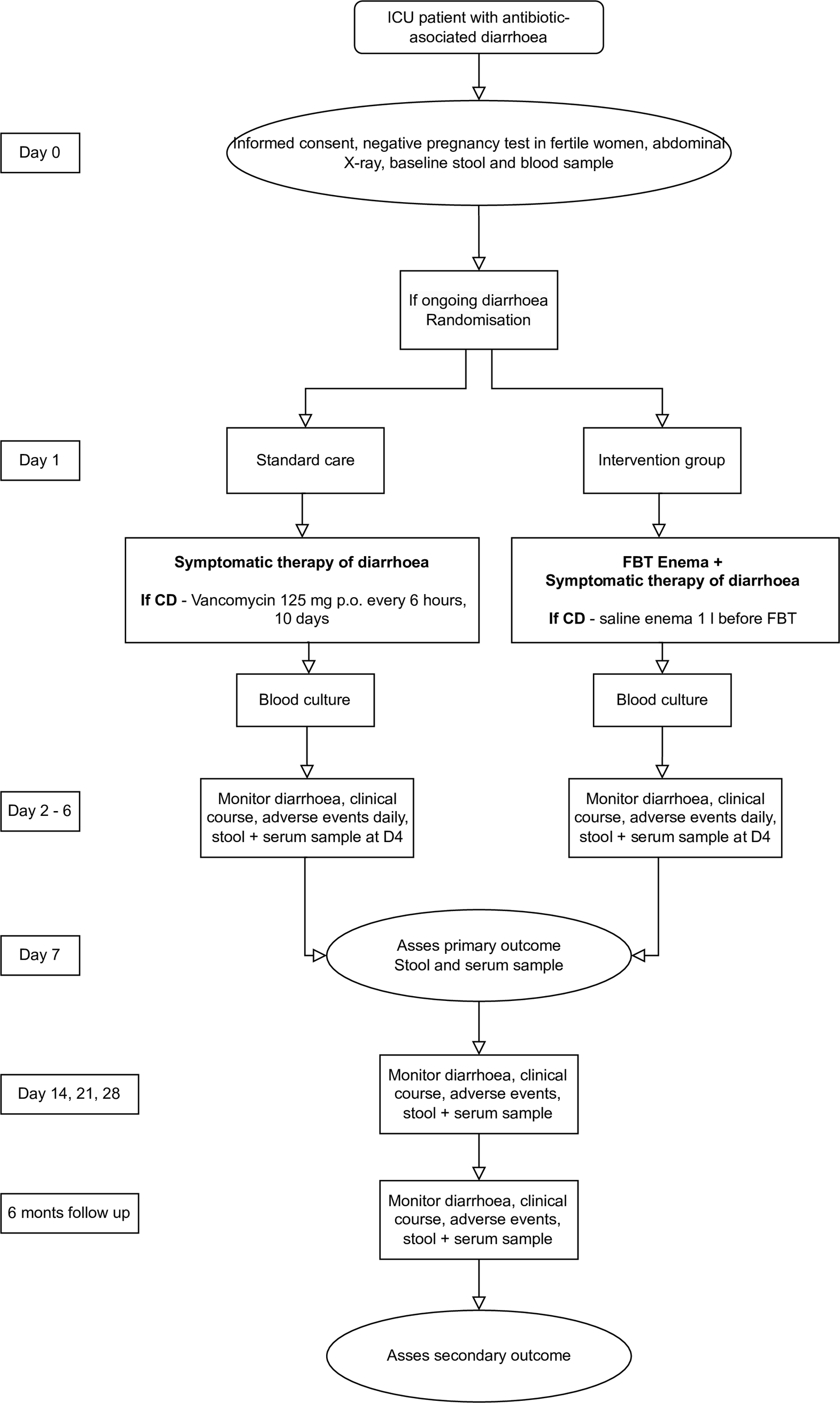

Supplement: S4 Appendix — (TIF) [file pone.0310180.s004.tif]
