# Supplementary material for: Allogenic faecal microbiota transplantation for antibiotic-associated diarrhoea in critically ill patients (FEBATRICE)–Study protocol for a multi-centre randomised controlled trial (phase II)
Source: PLoS One. 2024 Dec 27;19(12):e0310180. doi: 10.1371/journal.pone.0310180 (PMC11676529; doi:10.1371/journal.pone.0310180)
Supplement: S1 File — (DOCX) [file pone.0310180.s006.docx]

**Faecal Bacterial Transplantation for Postantibiotic Diarrhoea in Critically Ill Patients – Randomised Controlled Trial**

*Faecal Bacterial Transplant in Intensive Care*

ACRONYM: FEBATRIC

**Principal investigator:**

Dr Frantisek Duska, PhD, Dept. of Anaesthesia and Intensive Care Medicine, Charles University, 3^rd^ Faculty of Medicine and FNKV University Hospital in Prague, Czech Republic

**Investigators:**

Dr Veronika Rehorova – Dept. of Anaesthesia and Intensive Care Medicine, Charles University, 3^rd^ Faculty of Medicine and FNKV University Hospital, Prague

Dr Anne LeRoy – Dept. of Anaesthesia and Intensive Care Medicine, Charles University, 3^rd^ Faculty of Medicine and FNKV University Hospital, Prague

Dr Ivana Cibulkova – Division of Gastroenterology, Dept of Medidine II, Charles University, 3^rd^ Faculty of Medicine and FNKV University Hospital, Prague

Dr Jan Gojda - Centre for Research on Diabetes, Metabolism and Nutrition, Dept of Medidine II, Charles University, 3^rd^ Faculty of Medicine and FNKV University Hospital, Prague

Marek Kuzma, PhD – Institute of Microbiology, Academy of Sciences of the Czech Republic

Monika Cahova, PhD Department of Experimental Medicine, Institute for Clinical and Experimental Medicine, Prague

Dr Petr Waldauf - Dept. of Anaesthesia and Intensive Care Medicine, Charles University, 3^rd^ Faculty of Medicine and FNKV University Hospital, Prague

Dr Michal Fric - Dept. of Anaesthesia and Intensive Care Medicine, Charles University, 3^rd^ Faculty of Medicine and FNKV University Hospital, Prague

Dr Pavel Jansky - Dept. of Anaesthesia and Intensive Care Medicine, Charles University, 3^rd^ Faculty of Medicine and FNKV University Hospital, Prague

Dr Bob Bakalar - Dept. of Anaesthesia and Intensive Care Medicine and Burn Center, Charles University, 3^rd^ Faculty of Medicine and FNKV University Hospital, Prague

Dr. Robert Zajicek - Burn Center, Charles University, 3^rd^ Faculty of Medicine and FNKV University Hospital, Prague

Mrs Irena Kozakova, RN - Dept. of Anaesthesia and Intensive Care Medicine and Burn Center, Charles University, 3^rd^ Faculty of Medicine and FNKV University Hospital, Prague

Sponsor: Charles University, Third Faculty of Medicine, Progres Q37

Sponsor Responsible Representative: Dr Frantisek Duska, PhD, Dept. of Anaesthesia and Intensive Care Medicine, Charles University, 3^rd^ Faculty of Medicine and FNKV University Hospital in Prague, Czech Republic

Study monitor: Mrs Irena Kozakova, RN - Dept. of Anaesthesia and Intensive Care Medicine and Burn Center, Charles University, 3^rd^ Faculty of Medicine and FNKV University Hospital, Prague

Trial registration number (clinicaltrials.gov): **:**  NCT05430269

***Abstract:***

*Background:* Postantibiotic diarrhoea in critically ill patients is common, often protracted and currently there is no effective treatment or it.

*Aim*: To test safety and feasibility of faecal microbial transplantation in critically ill patients with postantibiotic diarhoea.

Design: Prospective, single center, parallel group randomised controlled trial.

Subjects: ICU patients (both general and burn ICU) who developed n critically ill patients who have diarhea after having finished a course of antibiotics that is persistent for 24 hours even after interruption of enteral nutrition. Patients with septic shock or approaching death will be excluded.

Treatment in the intervention group: Faecal microbial transplantation delivered as enema of 350 ml of standardised mixed transplantate prepared from faeces of 7 healthy donors.

Control group: Standard-of-care protocolised treatment of postantibiotic diarhea (which includes vancomycine 125 mg p.o. 6 hourly in the subgroup with C. dif. infection).

Primary outcome: Percentage of patients with treatment failure at day 7 after randomisation, which is defined as treatment either not being delivered or effective.

Secondary and exploratory outcomes: Influence of the intervention on colonic microbiom and metabolom, small bowel and colonic permeability, bacterial translocation and systemic inflammation.

***Background*:**

Over 80% of critically ill patients receive antibiotic treatment over their ICU stay and it is well documented that it leads in a reduction of the diversity of colonic bacterial flora. In turn, this leads to overgrowth of pathogenic species incl. Clostridium difficile (C.dif.) and multi-drug resistant Enterobacteriaceae and Enterococci. Restoration of physiological bacterial flora is prolonged and a significant proportion of patients suffer from postantibiotic diarrhoea. In particular, postantibiotic diarhea in the critically ill that is associated with C. dif. infection may lead to prolonged ICU stay or complications such as toxic megacolon, sepsis and death. Even non-clostridial postantibiotic diarhoea represent a significant burden to ICU patients and staff, impairing patients dignity and comfort and often increasing their length of stay. Current treatment options are limited as neither prebiotic nor probiotic were found to be consistently efficient in the critically ill and have been found risky in some settings, e.g. pancreatitis. Changes of colonic microbiome are well documented in the critically ill and length and mechanisms of restoration of colonic microbiome are yet unknown. It is also unknown to what extent changes in colonic flora contribute to nosocomial infections by MDR Gram negatives in the critically ill.

In the light of this, restoring physiological bacterial flora in the colon by faecal microbial transplant (FBT) could potentially be beneficial. FBT has been shown to be safe and effective not only in the prophylaxis of recurrence after the antibiotic treatment, but also as the first-line treatment in the population of non-critically ill patients with C. dif. infections.

The study will be performed in accordance with the protocol, The Declaration of Helsinki and principles of Good Clinical Practice: Consolidated Guidelines. The protocol is consistent with any applicable local regulations.

***Hypothesis and aims:***

We hypothesise that FMT will be feasible as first-line treatment of postantibiotic diarrhoea in the critically ill, incl. those with uncomplicated C. dif. infection.

*Primary end-point*:

Percentage of patients with treatment failure at day 7 after randomisation. Treatment failure is defined as either failure to administer allocated treatment for any reason or the presence of diarrhoea on day 7 after randomisation (as assessed by personnel blinded to treatment allocation).

*Secondary end-points are*:

- Composite number of adverse events such as new-onset sepsis, toxic megacolon, positive post FBT blood culture, or other SAE assessed by the physician-in-charge as possibly related to FBT.
- SOFA score at days 4 and 7.
- Percentage of patients that are recurrence-free at time of hospital discharge or day 28, whichever occur earlier.
- Subgroup analysis will be performed for patients who are C. dif. positive vs. negative and those who will receive antibiotics for a new extraabdominal infection vs. those who do not.

*Exploratory outcomes*:

- Diversity, density and composition of faecal microbiome by quantitative sequence-based mapping
- Changes in bowel metabolom incl. bile acid spectrum as assessed by NMR and GS/MS
- Bowel barrier integrity and inflammation assessed by plasma markers (citrulline, endotoxin, D-lactate, IL-6, IL-8, IL-12, TNF-a, LL-37 cathelidicine), faecal markers (calprotectine)

***Method:***

*Design*: prospective, randomised, controlled, parallel group, open-label, assessor blinded feasibility trial.

*Study subjects:*

- Inclusion Criteria: All of the following: Informed consent (see below), Age > 18 yrs., In-patient in ICU or HDU (incl. burn unit) and expected to stay for >7 days, Diarrhoea following antibiotic treatment defined as 3 or more stools per day or Bristol type 7 stool in the volume >300 ml/day if stool derivative device is in place, persisting for 24 hours despite enteral feeding formula has been stopped.
- Exclusion criteria: Death appears imminent or ceilings of care put in place, presence of new-onset sepsis defined as per 2016 definition, lactate >2.0 mM, colon diameter > 9 cm on plain AXR, the necessity of ongoing antibiotic treatment for another reasons. Unable to tolerate enema for any reason (e.g. surgery of the GI tract in the past year). Patients with a history of severe anaphylactic food allergy, any other reason which – as per judgement of the treating clinician – makes faecal transplantation unsafe or not feasible (Note: All screening failures based on this criterion will be reported separately, inc. the reason why it was considered unsafe or not feasible to proceed).

*Selection of donors and procedure of faecal graft preparation:*

The selection and assessment of donors will be performed after obtaining a written informed consent from them (see Appendix 1) and in accordance with Guidelines for faecal microbial transplantation (version May 2018, issued by Czech Society of Infectious Diseases, <https://www.infekce.cz/DPFMT18.htm>). The donors will be recruited mainly but not exclusively from volunteers donating their stool samples for another trial (Gojda et. al., NCT No), looking at the difference of intestinal microbioma between vegans and omnivores. We will only recruit donors into this study from the omnivorous cohort. After filling a pre-specified questionnaire (see Appendix 2), detailed history and physical examination will be performed. This will include examination of oral cavity and lymphatic nodes. Afterwards, the standardised donor work-up will include:

- Blood: full blood count incl. WBC differential, Sedimentation Rate, CRP, liver function tests (ALT, AST, ALP, GMT, bilirubin), creatinine, albumin and level of glucose , HIV (p24 and anti HIV-1 and 2), syphilis serology (RRR or TPPA) and hepatitis panel (HBsAg, anti-HBc IgM and IgG, HBeAg, anti-HBc total, anti-HCV, anti-HAV, anti-HEV)
- Stool examination: C.dif. (GDH and toxin A and B), cultivation to detect *Shigella, Campylobacter, Listeria, E.coli, Yersinia and Salmonella sp*., standard microscopy parasitological exam and from virological exam will be performed CMV PCR.

This examination is considered valid for 3 months, unless the donor developed any change or bowel habits, travelled abroad or taken antibiotics. Before each donation, a brief history will be taken to confirm this. For better timing of defecation, the donor will use glycerine suppositoria (Suppositoria Glycerini 2,06g). The stool will be captured via Fe-Col Faecal Collection paper (Eastoport) and transported in a sterile container (container 2000ml, Sarsted) to the place of processing as quicly as possible, but no later than 4 hours from defecation. The stool is weighted and mixed (hand blender, Mulinex) with normal saline 1:3, mixed thoroughly and filtered through 3 layers of gauze to remove solid parts. Afterwards, glycerine will be added to final concentration 10% and the final micture is divided into 50 ml sealed containers, labelled and immediately stored at -80 C for up to 6 months. In order to minimise the homogeneity of the transplantate, 2 hours before transplantation, 7 aliquotes from 7 donors will be thawed at 37C water bath and drawn into the irrigation syringes/bags.

*Sample size:*

We plan to randomise 36 patients in 1:1 ratio: This is a pilot trial, with primary outcome being feasibility and safety of faecal bacterial transplantation in the population of critically ill adults with C. dif. positivity and diarrhoea. However, with 36 randomised patients, the study will have sufficient power to determine non-inferiority against standard-of-care based on data available in FBT in patients with C. dif. In two trials in non-critically ill patients, the success of faecal transplantation was 69% (22 out of 32) or 56% (5 out of 9) as compared to 45% cured by oral metronidazole. Assuming that clinically significant difference between the two methods is 20% and the intervention will be as effective in ICU patients as it was in the non-critically ill group (curing 27 out of 41, ie. 66%), if there is a true difference in favour of the experimental treatment of 21%, then 36 patients are required to be 80% sure that the upper limit of a one-sided 95% confidence interval (or equivalently a 90% two-sided confidence interval) will exclude a difference in favour of the standard group of more than 20%. The expected duration of participation in the study is 6 months.

*Informed consent procedure:*

All patients with capacity will be asked to provide a prospective written informed consent (See Appendix 3). For patients without capacity, a deferred consent procedure will take a place. In this case, an independent clinician will review and sign that the patient is lacking capacity and he/she fulfils all criteria to be enrolled to the study. The patient’s next of kin will be informed about the study as soon as practical with the aid of information leaflet (Appendix 3). The patient will be asked to provide and sign informed consent as soon as he/she regains the capacity to do so. In case of consent refusal, patient’s data will not be used in per-protocol analysis.

Day 1

Follow up weekly until day 28 or hospital D/C

Monitor diarhea + clinical course daily, stool + serum sample at D4

Monitor diarhea + adverse events daily, stool + serum sample at D4

Perform FMT

(if C. dif. NO ATB therapy)

Treat diarrhoea per protocol

(if C. dif. add NS lavage + NG vancomycine 125 mg x4 /day)

STANDARD OF CARE

If ongoing diarhea, NS lavage, X-ray

RANDOMISE

Day 2-6

**Figure 1.: Simplified flowchart of study procedures. Note: BMS = Bowel management system (e.g. FlexiSeal ©); D/C= discharge; FMT = faecal microbial transplantation**

STANDARD OF CARE

Next day

*Procedures at baseline:*

**Day 0.** In patients with diarhea after obtaining informed consent, the drug chart will be reviewed and all medication that can be contributing to diarhea (e.g. metoclopramide) will be stopped if it is considered safe to do so. Abdominal X ray will be performed to rule out pneumoperitoneum or megacolon. After obtaining baseline stool sample (sent for study analyses but also for GDH and C. dif. toxins A and B, faecal calprotectin), blood sample will be tested for standard tests ( biochemistry, blood count, etc.). Stool and plasma samples will be frozen at -80 C for later analysis. Randomisation will be performed by sealed envelope in the evening of day No.0. Enteral nutrition will be stopped at 18:00 at the latest.

**Day 1:** The patient will be physically examined in the morning, including the determination of the degree of SOFA score (a set of laboratory results and examinations estimating organ function) and the frequency, amount and structure of the patient's stools will be recorded. Patients will be treated for their primary disease throughout the study according to established standard procedures.

In both groups adequate hydratation will be taken care of by replacing the fluids lost by diarrhea with infusion. Throughout the treatment, we monitor the patient’s vital functions (body temperature, heart rate, blood pressure, blood oxygen saturation)

- **Control group**: We will wait for spontaneus retreat of diarrhea, which is a standard of care. In the subset of patients with C. dif. positivity, the patients receive 125 mg of vancomycine 6 hourly (with rescue option of conversion to i.v. tigecycline).
- **Intervention group** :
- If considered safe, the patient receives Loperamide 2 mg p.o. 2 hours before FBT.
- FMT: 350 ml of thawed donor stool delivered by retention enema
- Faecal management system (e.g. FlexiSeal©) will be inserted and clamped for 2hours
- The patient will be positioned in left semilateral Trendelenburg's position for 15 minutes and then in right semilateral Trendelenburg’s position for another 15 minutes with faecal management device clamped.
- In all patients receiving FBT will be recorded body temperature, heart rate, oxygen saturation and blood pressure.
- In the subset of patients with C. dif. positivity, no antibiotics will be administered (with rescue option of administration of i.v. tigecycline).

**FBT procedure**:

The FBT suspension will be stored in specific −80°C freezer with connected alarm notification to guarantee continuous registration of the storage. Information on the FBT suspension labels includes donor code, suspension number, production and expiration date, volume, and storage temperature instruction. From each donor one of the sample will be store in freezer for the duration of the ongoing study.

The resulting faecal bacteriotherapy (FBT) preparation is defined as a mixture of 7 aliquots of processed stool intermediates from 7 donors.

The resulting graft will be drawn into four sterile 100 ml syringes and transferred to the ward where the recipient is hospitalized. Re-freezing of thawed or partially thawed extract is not permitted.

**Day 2, 3** Physical examination, SOFA score, evaluation of laboratory blood results (WBC, CRP, lactate, etc.) will be recorded as well as volume or frequency and consistency (Bristol scale) of stool. Concomitant medication and adverse events will be recorded.

**Day 4:** As above plus obtain stool + plasma samples, freeze -80 C

**Day 5, 6**  see Day 1, 2, 3

**Day 7:** Primary outcome assessment (see below) followed by collection of plasma and faecal samples.

*Assessment of primary outcome*:

An independent data analyst will retrospectively review the de-identified printouts of nursing notes from the two 12 hour shift day + night ending in the morning of day 7 and record the presence or absence of diarrhoea (defined as 3 or more stools per day or Bristol type 7 stool in the volume >300 ml/day if stool derivative device is inserted).

In case the patient is not on ICU, but remains at hospital, the research nurse will bring a special record form on day 6 and instruct both the patient and nursing staff to record during the next 24 hours the volume or frequency and consistency of stool using visual Bristol Scale and also to provide a sample of stool. In case the patient has been discharged, he/she will be contacted the day before over the phone by a research nurse and the next day the patient will be asked to bring the formulary and a stool sample to hospital of will be visited by a research staff at home.

Afterwards, as long as the patient remains on ICU, adverse events, antibiotic treatment, and stool volume/frequency are monitored daily.

In patients with faecal management device in place, follow up visits (identical to Day 4) are performed in 7 days interval up to day 28, and/or ICU discharge or FMD removal, whichever occurs earlier.

In patients who remain in ICU, but have had FMD removed, have the same follow up procedures, with the exception of stool sampling. Stool is collected and stored as close as practical to the day of the visit after the patient has had spontaneously defecated.

In patients who are not on ICU, but remain at hospital, will be visited by a research nurse at day 7, 14, 21 and 28 as long as they remain hospitalised. The visit will include monitoring of adverse event, notice on patient general status, the presence/absence of diarrhoea, antibiotic medication. Blood will be collected and frozen.

Six month follow up is performed at follow up clinics and it includes history (incl. recent history of diarrhoea), physical exam and blood sampling. Patients willing to do so are requested to bring in a stool sample. Patients unwilling to show up will be interviewed over

the phone.

|  | D0 | D1 | D2 | D3 | D4 | D 5-6 | D7 | D14, 21, 28or D/C | 6month follow up |
| --- | --- | --- | --- | --- | --- | --- | --- | --- | --- |
| ICF | x |  |  |  |  |  |  |  |  |
| Review drug chart | x |  |  |  |  |  |  |  |  |
| Serum sample + freeze | x |  |  |  | x |  | x | x | x |
| Stool sample + freez | x |  |  |  | x |  | x | (x) | x |
| Adverse events, Phys.exam, SOFA | x | x | x | x | x | x | x | x |  |
| Blood culture | x |  | x |  |  |  |  |  |  |
| Primary outcome assessment |  |  |  |  |  |  | x |  |  |

**Table: Gantt diagram of study procedures. See text for details.**

***Monitoring of safety:***

Although it is potentially beneficial and has been safely performed in ICU patients before [xx-xx], there is not enough data about FMT safety in patients with potentially disrupted intestinal barrier. In turn, following measures have been taken to maximise safety of the current study for patients:

- Treating clinician will not be blinded to treatment allocation and they will be encouraged to speak up should any safety concerns arise.
- Before and 3 hours after FMT, vital signs, blood cultures, endotoxin and early cytokine levels will be measures to specifically look at potential bacterial translocation.
- All patients’ vital functions will be continually monitored as per local standards. Frequency, volume and character (as per Bristol scale) of stools will be recorded in 12 hour intervals.
- Onset of sepsis or elevation lactate > 2mM or the presence of pyrexia >38 C with >30% rise of inflammatory markers without clear alternative reason (e.g. HAP) will trigger, in both groups, the addition of intravenous tigecycline or intravenous vancomycine, if C. dif positivity is known or suspected.
- I.v. antibiotics efficient against C. dif. can be added in both groups, or oral vancomycin or fidaxomycine can be added in the interventional group if a treating clinician considers this appropriate. Reason for doing so will be recorded.
- Should a SUSAR appear, it will be reported as per local legislature and within 48 hours reviewed by the team of investigators, together with independent safety advisors (prof. Jiri Benes. Dept. of Infectious Diseases, Na Bulovce Hospital, Prague and prof Jan Pachl, REB Chair FNKV Univ Hospital), a representative of nursing staff independent on the study. Two SUSARs in the intervention group would automatically lead to termination of the trial for safety reasons.
- In addition, the same team will meet ad hoc whenever safety concerns arise. The team will review data collected from first 8 subjects who have been randomised and focus on the analysis of adverse events. In particular, the team will review the dynamics of body temperature, heart rate, blood pressure, lactate and blood cultures before and after FMT. The decision will be taken to continue or terminate the study.

***Statistical plan:***

Safety parameters will be reported on intention-to-treat basis. Primary outcome will be compared by as unadjusted hazard ratio of not being cured (with 95% interval) in a per protocol analysis. The subset of patients with C. dif positivity will be analysed separately.

***Direct access to source data/documents:***

Whole FBT procedure must be properly recorded in accordance with the rules on medical records. The patient documentation shall include the performance indication, performance record, informed consent to FBT and information obtained during subsequent visits. The documentation must be kept to allow for an additional assessment of possible early and late side effects of the method. Furthermore, it will be recorded from what 7 donors the extract was used for each individual patient.

Donor documentation includes the results of clinical and laboratory testing, a completed and signed questionnaire confirming the absence of exclusion criteria and informed consent to the use faeces for medical purposes. For each frozen faecal extract, the date of freezing and, where appropriate, the place of preservation must be indicated in addition to the donor identification.

The examiner and the medical facility will allow for clinical trial monitoring, audits, oversight of the ethics committee, inspection authorities inspection and access to source documents.

***Quality control and quality assurance:***

To ensure compliance with Good Clinical Practices and all applicable regulatory requirement.

***Ethics:***

The final study protocol, including the final version of the Informed Consent Form, must be approved or given a favourable opinion in writing by in the Ethics Committee (EC) as appropriate. The Investigator is also responsible for informing the EC of any amendment to the protocol in accordance with local requirements. The investigator is also responsible for providing the EC with reports of any serious side effect of FBT therapy.

The study will be performed in accordance with the protocol, ethical principles that that have their origin in the Declaration of Helsinki WMA, and are consistent with Good Clinical Practice: Consolidated Guidelines and any applicable local regulation.

The Investigator(s) will ensure that subject (or the legal representative) is given full and adequate oral written information about the nature, purpose, possible risk and benefit of the study. Subject must also be notified that they are free to discontinue from the study any time. The subject (or the legal representative) should be given the opportunity to ask questions and allowed time to consider the information provided.

Voluntary written informed consent must be obtained before any study-related procedures are performed and the Investigator(s) must maintain the original, signed Informed Consent Form.

***Data handling and recordkeeping:***

The team of investigators, together with independent safety advisors (prof. Jiri Benes. Dept. of Infectious Diseases, Na Bulovce Hospital, Prague and prof Jan Pachl, REB Chair FNKV Univ Hospital) and representative of nursing staff independent on the study, are allowed to inspect subject charts, study source documents, drug accountability records and other records relative to study conduct. The Investigator must maintain all documentation to the study for a period of 5years after the end of last study subject.

***Publication Policy:***

We follow the authorship criteria established by the international Committee of medical Journal editors.

Minimum criteria for authorship credit based on: Substantial contributions to conception and design, acquisition of data or analysis and interpretation of data. Drafting the article or revising it critically for important intellectual content and final approval of version to be published. To be considered, all potential authors must fulfil all the minimum criteria for authorship credit.
