# Supplementary material for: Allogenic faecal microbiota transplantation for antibiotic-associated diarrhoea in critically ill patients (FEBATRICE)–Study protocol for a multi-centre randomised controlled trial (phase II)
Source: PLoS One. 2024 Dec 27;19(12):e0310180. doi: 10.1371/journal.pone.0310180 (PMC11676529; doi:10.1371/journal.pone.0310180)
Supplement: Attachment — Submitted filename: Response to Reviewers PLOS Protocol 1.2.docx [file pone.0310180.s007.docx]

Rebuttal letter to Cibulkova et. al.

Dear Editor,

We thank the reviewers for their insightful comments to the paper: *Allogenic faecal microbiota transplantation for antibiotic-associated diarrhoea in critically ill patients (FEBATRICE) – Study protocol for a multi-centre randomised controlled trial (Phase II).*

In this rebuttal letter, we provide step-by-step description of the changes we made in the revised manuscript.

I. **Response to journal requirements:**
1. *Please ensure that your manuscript meets PLOS ONE's style requirements, including those for file naming*

- We have corrected the manuscript as recommended. The changes are apparent in the manuscript in track-changes format.

2. *We note that the grant information you provided in the ‘Funding Information’ and ‘Financial Disclosure’ sections do not match. When you resubmit, please ensure that you provide the correct grant numbers for the awards you received for your study in the ‘Funding Information’ section.*

- We altered funding section of the main manuscript to make sure consistency with the appendices.

3. *When completing the data availability statement of the submission form, you indicated that you will make your data available on acceptance. We strongly recommend all authors decide on a data sharing plan before acceptance, as the process can be lengthy and hold up publication timelines. Please note that, though access restrictions are acceptable now, your entire data will need to be made freely accessible if your manuscript is accepted for publication. This policy applies to all data except where public deposition would breach compliance with the protocol approved by your research ethics board. If you are unable to adhere to our open data policy, please kindly revise your statement to explain your reasoning and we will seek the editor's input on an exemption. Please be assured that, once you have provided your new statement, the assessment of your exemption will not hold up the peer review process.*

- Thank you. We altered Data Management paragraph to include very specific data sharing plan for FEBATRICe study: “Data will be entered into a custom-made electronic Case Report Form (eCRF), accessible online, ensuring ease of input and consistent data capture across sites. To enhance data quality, range checks will be implemented to identify any out-of-range or inconsistent data values. All identifiable patient data will be stored on a secure hospital server. After the study's completion and following a six-month embargo period, deidentified datasets will be made available for secondary research using the REDCap (Research Electronic Data Capture) platform, a secure, web-based application designed to support data capture for research studies. Deidentification will involve removing or anonymizing all personal identifiers, including names, addresses, and other direct identifiers, as well as employing pseudonymization techniques for indirect identifiers. We will also use REDCap tools for access control and establish data-sharing agreements. The study's data management practices will comply with ethical guidelines and legal requirements, including GDPR, ensuring responsible data stewardship.”

4. *One of the noted authors is a group or consortium [FEBATRICE (Faecal Bacteriotherapy in Intensive Care) Consortium investigators ]. In addition to naming the author group, please list the individual authors and affiliations within this group in the acknowledgments section of your manuscript. Please also indicate clearly a lead author for this group along with a contact email address.*

- Thank you for noticing. We have corrected the title page and added an Acknowledgements section in the manuscript; the changes are listed in the revisions (line 6, line 540-543).

5. *We note that the original protocol that you have uploaded as a Supporting Information file contains an institutional logo. As this logo is likely copyrighted, we ask that you please remove it from this file and upload an updated version upon resubmission.*

-Thank you for the information. We have modified the documents (S2 Appendix – Donor Questionnaire, S3A Appendix – Informed Consent Form (Czech), S3B Appendix – Informed Consent Form (English Translation) as recommended. The logo of Donatio Intensivistam Endowment Fund is not copyrighted and has been kept.

6. *Please review your reference list to ensure that it is complete and correct. If you have cited papers that have been retracted, please include the rationale for doing so in the manuscript text, or remove these references and replace them with relevant current references. Any changes to the reference list should be mentioned in the rebuttal letter that accompanies your revised manuscript. If you need to cite a retracted article, indicate the article’s retracted status in the References list and also include a citation and full reference for the retraction notice.*

We have double checked the reference list. We do not cite any retracted papers.

II. **Response to reviewers comments:**

 Reviewer #1:

*Comment 1: My major concern was that I have some doubts about the choice of a time-to-event (TTE) analyses for an outcome where the follow up is only seven days. The time scale is never mentioned here. If it is days, then there may be many "tied" times which can be problematic for TTE analyses. Furthermore, the sample size appears to be based on the difference of two percentages and yet the primary analyses calls for a hazard ratio which requires a time-to-event analysis. The power analyses should match the methods used in the primary outcome analyses.*

- We 100% agree with this comment. Although there are 28days and 6 months follow ups planned in the study, determining exact times of event retrospectively might have been difficult and therefore, we changed plan for mortality analysis and hence, none of the prespecified outcomes is based on time-to-event analysis. Timing of primary outcome assessment of 7 days after the intervention was a pragmatic choice based on clinical practice, where most diarrhea caused by dysbiosis should resolve once the physiological diversity of the patient's gut microbiota is restored. Most importantly though, choosing longer interval would increase the proportion of patients who are discharged by this time, making objective diarrhea assessment difficult or impossible.

*Comment 2: I then found it odd that for an outcome with a 28-day follow up period, an odds ratio is called for (line 379). Somewhere in that paragraph (lines 378-382) it should be mentioned that logistic regression is being used. Kaplan-Meier curves might be more useful for this longer follow up…but why run a non-TTE analysis and then a TTE analysis? I'm not following the logic here.*

- Thank you for spotting this, we have removed time-to-event analyses incl. Kaplan-Meier comparisons for mortality from analyses plan – see also previous response.

*Comment 3: Finally, the majority of the protocol is written well enough, there are some instances of missing definite articles, singular forms used when plurals are correct, etc. The authors should note that PLOS ONE submissions are not copyedited before publication and manuscripts that don't meet the language standards can be rejected.*
*Specific comments:
1. (lines 154-155) This is not a complete sentence.*

- Thank you for your comment, we have corrected it in the revised manuscript (line 146-148) and also proofread the final version of the manuscript.

2. *Per the PLOS ONE study protocol guidelines, the aim of the study should be stated in the Materials and Methods section. The primary and secondary outcome sections come close, but I'm not sure that they quite capture the aims.*

- Thank you for your comment. We have added information about the aim and hypothesis to the Methods section in the revised manuscript (line 143-145)

3. *(lines 316-323) Please reference the package(s) in R or the randomization algorithm used to implement the randomization.*

- Thank you for your comment. We have added the requested information to the revised manuscript (line 313)

4. *(lines 383-391) It would be helpful to explain why each of these methods are being used. Some I am not familiar with, but others I know and am curious as to how you will use those methods.*

- The methods chosen for this study, including DESeq2 normalization, vegan PERMANOVA, and Lasso/Ridge regression using glmnet, were selected for their specific strengths in analyzing microbiome data and addressing the complex statistical needs of the study. When preparing the manuscript, we wandered to what depth/detail explanations should go. We have opted for brevity, assuming that microbiome scientist would understand this. Nonetheless, we agree with the reviewer, that this choice might not be clear enough for a general readership. Therefore, to each method we added a footnote, explaining them and also briefly the rationale for their use. We are also happy to move this information to the Supplement or manuscript body.

5. (lines 408-409) The methods for stopping for futility should be articulated here or in supplementary materials.

- Thank you for this comment. Since this is a phase II trial planned on 36 subjects which aims to gather feasibility and microbiome biology data, there are no pre-defined stopping rules based on futility, the study will only stop for safety concerns. This is now clearly stated in the revised manuscript.

Reviewer #2:

1. *What is HDU (line 163)?*

- Thank you for your comment. We added the abbreviation "HDU" (high dependency unit) to the list of abbreviations at the end of the manuscript

2. *I suppose that "inc." (line 188) means "including", but "incl." (line 196) is also "including"?*

- Thank you for your comment. We wrote the different abbreviations in full in the text of the manuscript ( line 171, 184)

3. *Line 203. Please correct "for one our" to "for one hour"*

- Thank you for your comment. We have corrected an error in the text (line 199)

4. *Lines 207, 228, 301. Please correct "clostrioides" to "clostridioides"*

- Thank you for your comment. We have corrected errors in the text (line 203, 224, 258, 260, 281, 295)

Thanks again for your time and effort to improve our manuscript. We sincerely hope that the manuscript in revised form will now be suitable for publication in PloS-One.

Best regards

Frantisek Duska – on behalf of the authors.
